# Intended preoperative trans-arterial embolization for large hepatocellular carcinoma: a retrospective cohort study

**DOI:** 10.1186/s12957-022-02563-9

**Published:** 2022-03-22

**Authors:** Ryo Saito, Hidetake Amemiya, Naohiro Hosomura, Hiromichi Kawaida, Katsutoshi Shoda, Shinji Furuya, Hidenori Akaike, Yoshihiko Kawaguchi, Shingo Inoue, Hiroshi Kono, Daisuke Ichikawa

**Affiliations:** First Department of Surgery, Faculty of Medicine, University of Yamanashi, 1110 Shimokato, Chuo, Yamanashi, 4093898 Japan

**Keywords:** Trans-arterial embolization, Hepatectomy, Hepatocellular carcinoma, Large tumor

## Abstract

**Background:**

Generally, a large tumor size of hepatocellular carcinoma (HCC) is associated with poor visibility and uncertainty in the surgical field which results in increased surgical difficulty as well as unfavorable postoperative outcomes. We performed intended preoperative trans-arterial embolization (TAE) in patients with a large HCC. In this study, we investigated the oncological significance of intended preoperative TAE for a large HCC, using a comparison between patients with and without TAE, and detailed analyses for pre- and post-TAE status.

**Methods:**

A total of 411 patients who underwent hepatectomy for primary HCC at the University of Yamanashi Hospital between January 2007 and December 2018 were included in this study. The patients were divided into two groups: patients with larger HCCs (≥50 mm, *n*=51) and those with smaller HCC (<50 mm, *n*=360) according to the size of their HCCs. Comparison of clinicopathological features between these groups and clinical outcomes between the TAE and non-TAE groups were compared. In addition, a detailed analysis of each case in the TAE group was conducted, comparing clinicopathological factors between pre- and post-TAE status.

**Results:**

The clinical unfavorable short- and long-term outcomes of patients with large HCCs (≥50 mm) were revealed compared to those with small HCCs (<50 mm). The prognostic analyses showed that a large tumor size and increased tumor markers, multiple tumor numbers, and others were adverse prognostic factors, and vascular invasions and residual tumors were included in the multivariate analysis.

Further detailed analyses revealed that the average rates of change in tumor size and tumor shrinkage after TAE were − 48.6±35.6 mm and − 30.7±17.0%, respectively. Pathological high necrotic changes in the tumor, after multiple-times TAE aiming to a better effect, were related to a better prognosis in patients with large HCC. Poor prognostic factors became less common in patients who underwent intended preoperative TAE, and these patients had better prognoses.

**Conclusions:**

The large tumor size of HCC is associated with unfavorable outcomes; the intended preoperative TAE for large HCC patients performed multiple times aiming to affect the tumor as much as possible might improve their prognoses.

## Background

Liver cancer is one of the major malignancies and ranks (2020) sixth in incidence and third in mortality worldwide [1]; hepatocellular carcinoma (HCC) accounts for more than 90% of the cases of liver cancer [2]. HCC generally occurs due to chronic and continuous inflammation caused due to hepatitis B or C virus (HBV or HCV), alcoholic hepatitis, or non-alcoholic steatohepatitis (NASH). Of these, HBV and HCV are well-known cancer-associated viruses and are investigated as therapeutic targets; patients infected with HBV and HCV are being followed up to maintain surveillance on their status of hepatitis and cancer occurrences using blood tests and imaging examinations. In contrast to these hepatitis virus-induced HCCs, non-B non-C (NBNC) HCCs, caused mainly due to alcoholic hepatitis and NASH, have been growing in recent years [3]. HCCs developing in these NBNC patients are generally detected at a relatively advanced stage due to the lack of follow-up or medical examinations [4]. Thus, the demand for the establishment of treatment strategies and improvement of clinical outcomes for large (≥50 mm) or huge (≥100 mm) HCCs has been increasing.

Generally, a large tumor is associated with poor visibility and uncertainty in the surgical field which results in increased surgical difficulty [5]. In addition, recent studies have revealed that postoperative complications also lead to poor long-term outcomes in various malignancies [6–8]. Therefore, additional manipulations should be considered to improve the short- and long-term outcomes in patients with large HCCs. We situationally performed the intended preoperative trans-arterial embolization (TAE) for patients with large HCC as a preoperative neoadjuvant therapy for patients with resectable tumors, aiming for oncologically more curability. In this study, we have revealed the clinicopathological differences between patients with large or small HCCs and have evaluated prognostic factors in patients with HCC. Furthermore, we investigated the oncological significance of the intended preoperative TAE for a large HCC using a comparison between patients with and without TAE; detailed analyses for pre- and post-TAE status were also performed.

## Materials and methods

### Patients and ethical concern

A total of 411 patients who underwent hepatectomy for primary HCC at the University of Yamanashi Hospital between January 2007 and December 2018 were included in this study. Patients with non-HCC tumors (intrahepatic cholangiocarcinoma, metastatic tumor, or others) were excluded. This study was approved by the Ethics Committee of the University of Yamanashi (approval number, 2050) and was performed in accordance with the ethical standards of the Declaration of Helsinki and its later amendments [9].

### Evaluation of patients’ and tumors’ factors and perioperative outcomes

The reserved liver functions and blood examination, including tumor markers such as alpha-fetoprotein (AFP), the L3 fraction of AFP (AFP-L3), and protein induced by vitamin K absence or antagonist II (PIVKA-II), were indicated as pre-treatment (pre-TAE and preoperative) data. Reserved liver functions were evaluated using the Child-Pugh classification and indocyanine green (ICG) retention test. Hepatitis was classified as HBV, HCV, NBNC including alcoholic hepatitis and NASH, and others. Tumor sizes were evaluated based on pathological findings of resected tumors, while preoperative tumor size was measured using computed tomography (CT) imaging. Clinical data, including laboratory examination results, postoperative prognoses, and operative outcomes, such as intraoperative blood loss (IBL) and operative time, were obtained from the electronic medical recording system at the University of Yamanashi Hospital.

### Indication criteria of preoperative TAE and portal vein embolization

After evaluation of general and tumor statuses using blood tests and imaging examinations, huge HCCs were revealed in several patients; these were perceived by hepatology surgeons as considerably difficult surgical procedures with a risk of a large amount of IBL and/or deficient volume of the remnant liver. Therefore, these patients underwent the intended preoperative TAE for the purpose of tumor shrinkage and of facilitating difficult surgical procedures because of the large tumor size. TAE manipulation was performed by professional radiologists using conventional methods with lipiodol and/or antitumoral agents. The therapeutic effects of TAE were evaluated using contrast CT examination and blood tests. TAE procedures were practiced repeatedly to achieve a sufficient effect against tumor viability. In addition, with or without preoperative TAE, some patients who were assumed to undergo extended liver resection were pretreated with portal vein embolization. After these intended preoperative procedures, surgical resections were performed for patients with large HCCs.

### Postoperative following up protocol and prognostic analyses

Postoperative follow-up was performed with evaluation of tumor markers every 3 months and imaging examination by contrast CT or magnetic resonance imaging and/or complementary abdominal ultrasonography every 3 to 6 months [10]. Recurrence of HCC was determined by newly detected recurrent tumors based on imaging examination or by the obvious hypervascularity of the already known early HCCs. Patients were treated for recurrent tumors by re-resection or other methods, such as radiofrequency ablation, TAE, or systemic chemotherapy, according to tumor and patient conditions. Postoperative prognoses were evaluated by 2-year recurrence-free survival (RFS) and 5-year disease-specific survival (DSS).

### Three steps of analyses to evaluate the significance of preoperative TAE

To evaluate the significance of preoperative TAE, three steps of analysis were conducted in this study. First, the patients were divided into two groups: group L (with larger HCC; ≥50 mm, *n*=51) and group S (with smaller HCC; <50 mm, *n*=360) according to the size of their HCCs. The patients’ background, including age, sex, reserved liver function and hepatitis etiologies, and tumor-associated factors such as tumor markers, tumor number (single or multiple), status of vascular invasions, and perioperative outcomes, were compared between the two groups. Furthermore, univariate and accompanied multivariate prognostic analyses for 5-year DSS were performed.

Second, the therapeutic outcomes of the intended preoperative TAE were evaluated using a large HCC cohort (51 patients). Among them, five patients had undergone preoperative TAE aimed at tumor shrinkage and/or oncological effect (TAE group). Comparative analysis between patients in the TAE group and those without preoperative TAE (non-TAE group), particularly focusing on the aforementioned prognostic factors in the first prognostic analysis, and prognostic analyses for 2-year RFS along with 5-year DSS were performed. Finally, a detailed analysis of each case in the TAE group was conducted. Changes in tumor size, laboratory examination, relationship between frequency of TAE, pathological necrosis rate, and prognosis were evaluated.

### Statistical analysis

All continuous variables have been represented as median values and their ranges or mean ± standard errors, unless noted otherwise. Statistical analyses were performed using the chi-square test and Student’s *t*-test. Univariate prognostic analyses were performed using the Kaplan–Meier method and log-rank test. A multivariate prognostic analysis was conducted using factors that were indicated with a *p* value of less than 0.1 according to the results of the univariate analysis. All statistical analyses were performed using EZR (Saitama Medical Center, Jichi Medical University, Saitama, Japan), a graphical user interface for R (The R Foundation for Statistical Computing, Vienna, Austria) [11]. Statistical significance was set at *p*<0.05.

## Results

### Clinicopathological features and short- and long-term outcomes for patients with large tumor size

**Fig. 1:**
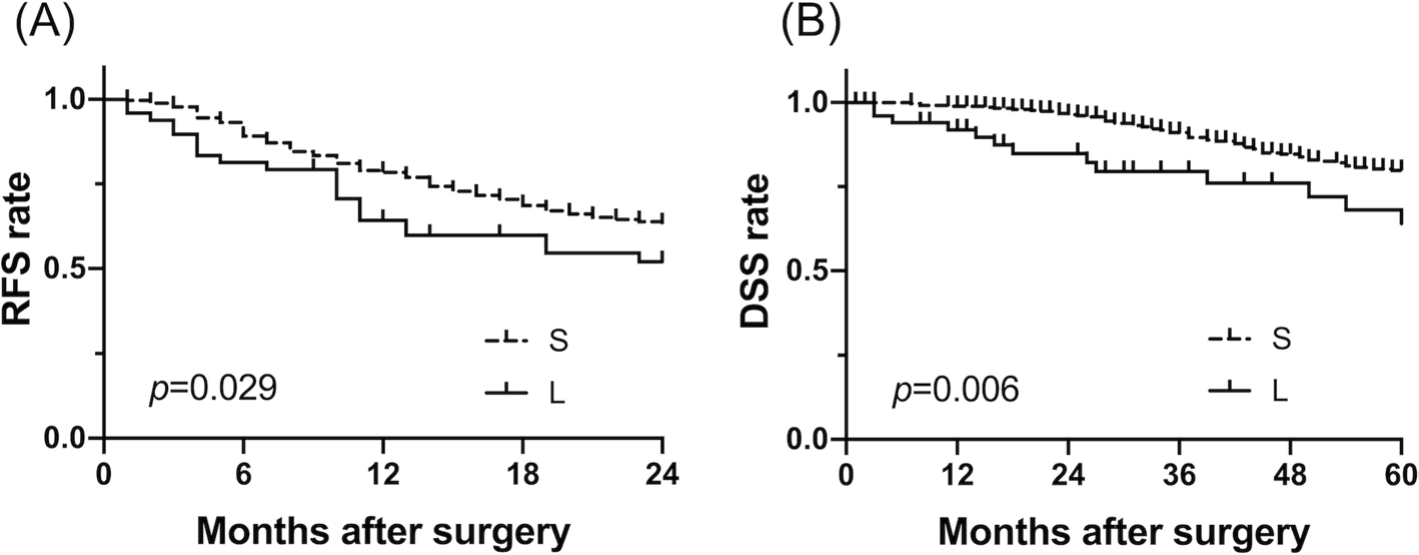
Prognostic analyses for 2-year RFS and 5-year DSS based on the tumor size. Prognostic analyses based on tumor size revealed that patients with large HCC (group L) showed significantly worse prognoses in the 2-year RFS (*p*=0.029) and 5-year DSS (*p*=0.006), compared to those with small HCC (group S). RFS, recurrence-free survival; DSS, disease-specific survival; HCC, hepatocellular carcinoma

The results of the comparison for all patients in groups L and S are shown in Table 1. More patients with NBNC hepatitis were included in group L, in contrast to group S, in which HCV was the dominant cause of background hepatitis in patients. As an inevitable consequence, large tumor size was related to high tumor marker levels and tumor vascular invasion for tumor factors. In terms of surgical procedure outcomes, patients in group L had significantly higher IBL and longer operative time as well as a higher frequency of perioperative blood transfusion. Subsequently, a prognostic analysis for 5-year DSS was performed using various clinical factors (Table 2). The results demonstrated that liver dysfunction in patients (Child-Pugh B), high levels of tumor markers, presence of lymph node metastasis, and others were significant adverse prognostic factors apart from the large tumor size (Fig. 1). Furthermore, the presence of vascular invasion, residual tumor (microscopic or macroscopic), and perioperative blood transfusion were indicated as independent adverse prognostic factors after multivariate analysis.

**Table 1:** Comparison of clinicopathological factors between L and S groups

Variable		S (<50 mm) ***n***=360 (%)	L (≥50 mm) ***n***=51 (%)	***p*** value
***Patient factors***				
**Age**		71 [35–89]	71 [16–85]	0.964
**Sex**	Male	289 (79.8)	42 (82.4)	0.851
**HBV infection**	Present	54 (15.0)	11 (21.6)	0.223
**HCV infection**	Present	202 (56.1)	13 (25.5)	**<0.001**
**ICG-R15 (%)**		14.6 [3.2–49.7]	13.3 [2.7–75.6]	0.251
**Child-Pugh**	B	11 (3.1)	2 (4.1)	0.665
***Tumor factors***				
**AFP (ng/ml)**		6.8 [0.5–13310.0]	11.0 [0.80–128900.0]	**0.009**
**AFP-L3 (%)**		0.0 [0.0–99.5]	7.2 [0.0–84.0]	**0.001**
**PIVKA-II (mAU/ml)**		24.0 [8.0–17483.0]	1011.0 [12.0–96988.0]	**<0.001**
**Tumor number**	Multiple	109 (30.1)	15 (29.4)	0.999
**Vascular invasions**	Present	68 (18.9)	26 (53.1)	**<0.001**
**LN metastasis**	Present	2 (0.7)	1 (2.0)	0.397
***Perioperative factors***				
**Preoperative treatment**^a^	Present	19 (5.2)	14 (27.5)	**<0.001**
**IBL (ml)**		531 [1–15759]	828 [122–3330]	**0.003**
**Operation time (min)**		375 [91–867]	467 [257–791]	**<0.001**
**Blood transfusion**	Present	49 (13.6)	13 (25.5)	**0.036**
**Residual tumor**	R1/2	12 (3.3)	5 (9.8)	**0.047**
**Postoperative complications**	CD ≥2	120 (33.3)	16 (31.4)	0.874

Consequent values are expressed as median and range

^a^Preoperative treatment means TAE and portal vein embolization

*HBV* hepatitis B virus, *HCV* hepatitis C virus, *ICG-R15* indocyanine green retention15, *AFP* alpha-fetoprotein, *AFP-L3* L3 fraction of alfa-fetoprotein, *PIVKA-II* protein-induced vitamin K absence or antagonist-II, *LN* lymph node, *IBL* intraoperative blood loss, *CD* Clavien-Dindo classification *TAE* trans-arterial embolization

**Table 2:** Univariate and multivariate prognostic analysis for disease-specific survival among all patients

Variable	Indicator	Univariate	Multivariate			
		***p*** value	HR	95% CI (range)		***p*** value
**Age**	≥70 (vs <70)	0.123				
**Sex**	Female (vs male)	0.161				
**HBV infection**	Present (vs absent)	0.214				
**HCV infection**	Present (vs absent)	0.255				
**Cirrhosis**	Present (vs Absent)	0.054	1.088	0.6123	1.935	0.773
**Child-Pugh**	B (vs A)	**<0.001**	1.019	0.3546	2.928	0.972
**AFP (ng/ml)**	≥10 (vs <10)	**0.005**	0.929	0.4952	1.743	0.819
**AFP-L3 (%)**	≥10 (vs <10)	**0.028**	1.595	0.8469	3.003	0.148
**PIVKA-II (mAU/ml)**	≥40 (vs <40)	**<0.001**	1.645	0.9274	2.919	0.089
**Tumor number**	Multiple (vs aingle)	**0.022**	1.206	0.7079	2.055	0.491
**Tumor size (mm)**	≥50 (vs <50)	**0.006**	0.7701	0.3534	1.678	0.511
**LN metastasis**	Present (vs absent)	**<0.001**	3.345	0.7132	15.69	0.126
**Vascular invasions**	Present (vs absent)	**<0.001**	2.125	1.234	3.659	**0.007**
**Differentiation**	Por/undif (vs well/mod)	**0.003**	1.358	0.7725	2.388	0.288
**Residual tumor**	R1/2 (vs R0)	**<0.001**	5.986	2.366	15.14	**<0.001**
**Postoperative complications**	≥CD2 (vs ≤CD1)	**0.022**	1.417	0.816	2.462	0.216
**Blood transfusion**	Present (vs absent)	**<0.001**	2.502	1.355	4.623	**0.003**

*HR* hazard ratio, *CI* confidence interval, *HBV* hepatitis B virus, *HCV* hepatitis C virus, *AFP* alpha-fetoprotein, *AFP-L3* L3 fraction of alpha-fetoprotein, *PIVKA-II* protein-induced vitamin K absence or antagonist-II, *LN* lymph node, *Por* poorly differentiated, *Undif* undifferentiated, *Well* well-differentiated, *Mod* moderately differentiated, *CD* Clavien-Dindo classification

### Evaluation for tumor-shrinking and oncological effects of intended preoperative TAE

**Fig. 2:**
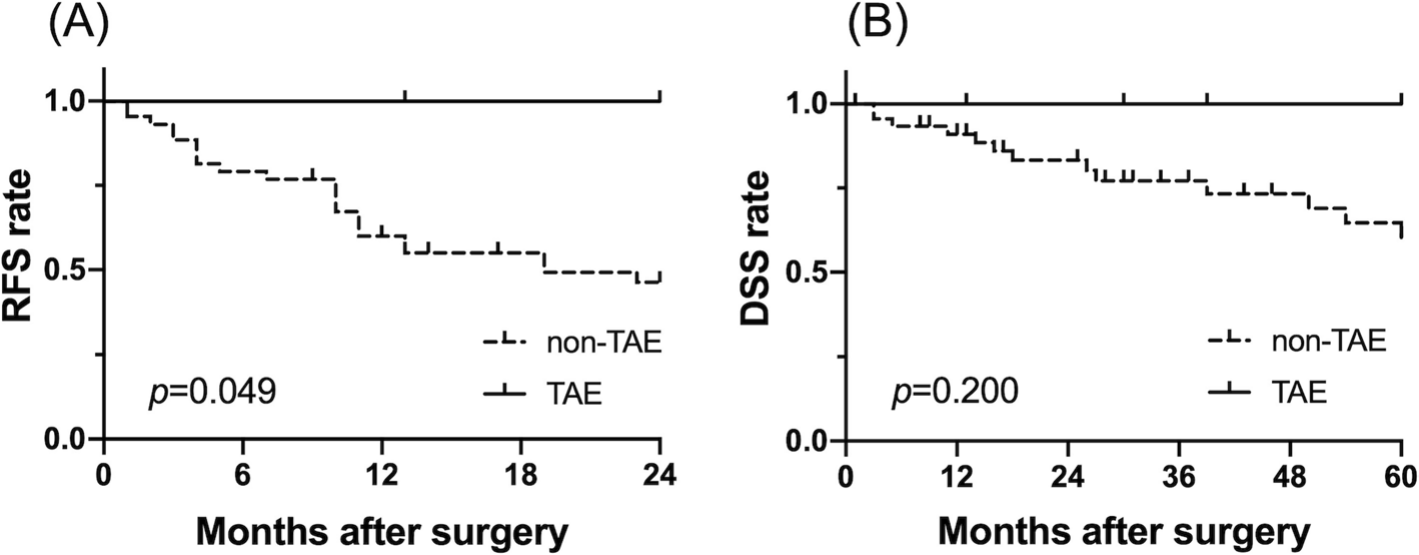
Prognostic analyses for TAE and non-TAE groups. Patients treated with the intended preoperative TAE showed relatively better prognoses in the 2-year RFS (*p*=0.049) and 5-year DSS (*p*=0.200), compared to those without preoperative TAE. RFS, recurrence-free survival; DSS, disease-specific survival; TAE, trans-arterial embolization

Comparison of characteristics between the two groups (TAE and non-TAE groups) demonstrated that patients in the TAE group had no residual tumor and had relatively lower tumor markers which were indicated as prognostic factors in the aforementioned univariate and multivariate prognostic analyses (Table 3). In fact, a significant or relatively better prognosis was shown in the TAE group for 2-year RFS (Fig. 2a, *p*=0.049) and 5-year DSS (Fig. 2b, *p*=0.200), respectively. However, the results also showed that there was still a certain level of difference in tumor sizes between the two groups even after the intended preoperative TAE. Due to such differences, patients in the TAE group underwent longer operations which were associated with higher IBL and blood transfusion; this was also demonstrated as a poor prognostic factor in the previous analysis.

**Table 3:** Comparison of characteristics between TAE and non-TAE groups

Variable		TAE (***n***=5) (%)	non-TAE (***n***=46) (%)	***p*** value
***Patient factors***				
**Age**		65 [63–78]	71 [16–85]	0.526
**Sex**	Male	5 (100.0)	37 (80.4)	0.571
**Etiology**	NBNC	3 (60.0)	22 (47.8)	0.999
**ICG-R15 (%)**		15.4 [7.5–24.8]	13.2 [2.7–75.6]	0.727
**Child-Pugh**	B	0 (0.0)	2 (4.5)	0.999
***Tumor factors***				
**AFP (ng/ml)**		7.2 [2.2–79.6]	14.8 [0.8–128900]	0.265
**AFP-L3 (%)**		0.0 [0.0–11.4]	9.9 [0.0–84.0]	0.150
**PIVKA-II (mAU/ml)**		125.0 [14.0–68901.0]	1056.0 [12.0–96988.0]	0.974
**Tumor size (mm)**		100 [55–130]	65 [50–140]	0.050
**Tumor number**	Multiple	3 (60.0)	12 (26.1)	0.144
**Tumor location**	Right lobe	5 (100.0)	26 (56.5)	0.143
***Perioperative factors***				
**PVE**	Present	2 (40.0)	7 (15.2)	0.209
**IBL (mL)**		2017 [828–2087]	717 [122–3330]	**0.011**
**Operation time (min)**		690 [629–791]	453 [257–694]	**0.001**
**Blood transfusion**	Present	4 (80.0)	9 (19.6)	**0.012**
**Residual tumor**	R1/2	0 (0.0)	5 (10.9)	0.999
**Postoperative complications**	CD ≥2	3 (60.0)	13 (28.3)	0.309

Consequent values are expressed as median and range

*TAE* trans-arterial embolization, *NBNC* non-B non-C hepatitis, *ICG-R15* indocyanine green retention15, *AFP* alpha-fetoprotein, *AFP-L3* L3 fraction of alfa-fetoprotein, *PIVKA-II* protein-induced vitamin K absence or antagonist-II, *PVE* portal vein embolization, *IBL* intraoperative blood loss *CD* Clavien-Dindo classification

### Detailed analyses for each case with intended preoperative TAE

Table 4 shows the detailed data of each patient who received the intended preoperative TAE for their huge HCCs. All cases had HCCs in the right lobe of the liver, and the tumor size was over 100 mm. The average rates of change in tumor size and tumor shrinkage were − 48.6±35.6 mm and − 30.7±17.0%, respectively. Except for case 1, TAE was repeated two or three times for each patient. In such cases, a higher necrotic rate was observed in the postoperative pathological findings compared to that with just one TAE. The changes in the laboratory examination data are shown in Table 5. Increased transaminases and unnaturally upregulated platelet counts improved and returned to normal levels after TAE. Variables reflecting reserved liver function, such as albumin, prothrombin time-international normalized ratio, and ICG retention rate at 15 min, were the same as before TAE. In contrast, markedly elevated tumor markers before TAE, such as AFP and PIVKA-II, were remedied after TAE. As a combined result of improvement of preoperative liver conditions and oncological effects for huge HCCs, no patients with intended preoperative TAE developed postoperative early recurrence (within 2 years) and HCC-specific deaths within 5 years, in contrast to those without preoperative TAE (Fig. 2).

**Table 4:** Detailed data of patients treated with preoperative TAE

Case	Age	Sex	Etiology	Tumor location	Tumor size (mm)	Frequency of TAE	Time to surgery (month)	Shrinkage rate (%)	Necrosis rate (%)	Recurrence	RFS (month)	Disease-specific death	DSS (month)
**1**	63	M	Alc	AP	110	1	3.2	− 22.7	40	+	40	-	137
**2**	71	M	Alc	AP	150	3	7.4	− 23.3	90	+	83	-	99
**3**	63	M	HCV	AP	240	3	5.1	− 45.8	90	-	39	-	39
**4**	78	M	HBV	P	120	3	6.4	− 54.2	100	-	25	-	25
**5**	65	M	Alc	PA	110	2	11.9	− 7.4	100	-	9	-	9

Tumor size is pre-TAE value

*TAE* trans-arterial embolization, *M* male, *Alc* alcoholic hepatitis, *HCV* hepatitis C virus-induced hepatitis, *HBV* hepatitis B virus-induced hepatitis, *A* anterior segment, *P* posterior segment, *RFS* recurrence-free survival, *DSS* disease-specific survival

**Table 5:** The changes of laboratory examination data between pre- and post-TAE status

Variable		pre-TAE ***n***=5 (%)	post-TAE ***n***=5 (%)	***p*** value
**AST (IU/l)**		73.8 ± 28.0	32.2 ± 13.5	**0.017**
**ALT (IU/l)**		44.2 ± 20.4	27.8 ± 14.3	0.180
**Total Bilirubin (mg/dl)**		0.74 ± 0.36	0.54 ± 0.29	0.359
**Albumin (g/dl)**		3.80 ± 0.77	3.84 ± 0.15	0.913
**PT-INR**		1.08 ± 0.07	1.08 ± 0.05	0.959
**Platelet count (/μl)**		37.8 ± 14.1	21.9 ± 6.0	**0.049**
**ICG-R15 (%)**		13.6 ± 2.6	14.9 ± 6.3	0.676
**Child-Pugh**	A	5 (100.0)	5 (100.0)	0.999
**AFP (ng/ml)**		2907.2 ± 6313.9	20.5 ± 33.2	0.337
**AFP-L3 (%)**		2.5 ± 4.6	3.7 ± 5.3	0.719
**PIVKA-II (mAU/ml)**		43019.0 ± 29839.0	5142.0 ± 10622.0	**0.028**

Consequent values are expressed as means ± standard errors

*TAE* trans-arterial embolization, *AST* aspartate aminotransferase, *ALT* alanine aminotransferase, *PT-INR* prothrombin time-international normalized ratio, *ICG-R15* indocyanine green retention15, *AFP* alpha-fetoprotein, *AFP-L3* L3 fraction of alpha-fetoprotein, *PIVKA-II* protein-induced vitamin K absence or antagonist-II

## Discussion

We noted clinically unfavorable short- and long-term outcomes in patients with large HCCs (≥50 mm) compared to those with small HCCs (<50 mm). Prognostic analyses showed that a large tumor size and increased tumor markers and multiple tumor numbers were adverse prognostic factors; vascular invasions and residual tumors were then included in the multivariate analysis. Further detailed analyses revealed that these factors became less common in patients who underwent intended preoperative TAE and had better prognoses. Thus, we have provided the oncological significance of the intended preoperative TAE for patients with large HCCs.

For gastrointestinal cancers including HCC, as well as other malignancies, both surgical resection and perioperative treatments have been more important along with improved treatment effects [12–15], and additional preoperative treatment has recently attracted more attention than postoperative treatment because of its superior tolerability resulting in a better general condition of the patients [16]. However, the adverse effects of preoperative treatment and insufficient general conditions of patients after aggressive preoperative intervention may become a problem at the time of highly invasive surgery. Thus, consideration in the selection of the intended preoperative treatment strategy especially for patients with resectable tumors is necessary. Percutaneous radiofrequency ablation (RFA), TAE, and surgical resection have been traditionally performed as local therapies in contrast to systemic chemotherapies [10]. In addition, it has also been recently reported that a combination of these local therapies, and/or systemic chemotherapies, can provide a higher therapeutic effect [17–19]. Therefore, HCC differs from other gastrointestinal cancers in that local treatments other than surgical resection have shown a certain therapeutic effect. Various treatment strategies are recommended according to the tumor factors and general patient condition (including reserved liver function) as per the Japanese clinical guidelines for HCC [10]. These local therapies, such as TAE, would be potential options as intended preoperative treatments for large HCCs.

HCCs show different recurrence patterns compared to other gastrointestinal malignancies—HCC is more likely to show intrahepatic metastases than systemic distant metastases, and intrahepatic recurrences are divided into two detailed patterns. One is multi-centric occurrence based on chronic hepatitis, and the other is intrahepatic metastatic recurrence from the primary HCC which mainly occurs through the intrahepatic portal veins [20]. In fact, para-tumoral daughter nodules are sometimes detected in highly advanced HCCs, as an example of intrahepatic and trans-portal metastases [21]. Tumor cells squeezed out from the primary tumor during surgery have also been shown to increase intrahepatic metastases. As for hepatectomy, especially for huge HCCs occupying the right lobe, severe displacement and mobilization of the liver, including a tumor, are necessary to keep the surgical field clear and prevent extreme IBL. The ironic truth is that this procedure may lead to intrahepatic seeding of tumor cells, which could lead to metastases. To prevent this, tumor necrosis as a result of preoperative TAE might be effective in enhancing surgical maneuverability and for suppressing trans-portal metastasis.

The prognostic significance of preoperative TAE has been well investigated in the 1990s and the 2000s. Some studies have suggested a favorable effect for patients with HCCs; however, most researchers did not agree with the positive results and concluded that preoperative TAE was not performed uniformly. One reasonable explanation for these unfavorable results might be the activation of tumor cells due to hypo-oxygenic stimulation caused by the insufficient therapeutic effects of TAE. In fact, Sasaki et al. suggested that preoperative TAE should be avoided for these patients, especially those without cirrhosis or with an early-stage tumor; however, most of the patients included in the study underwent only a single embolization [22]. In contrast, Zhang Z et al. [23] revealed that preoperative TAE performed multiple times suppressed HCC recurrence, and Lu et al. [24] and Sugo et al. [25] suggested that targeted preoperative TAE for patients at high risk of recurrence, such as large size or multiple tumor numbers, improved postoperative prognoses for HCC patients. In our study, we found that pathological high necrotic changes in the tumor were related to a better prognosis in patients with large HCC; this was not achieved in a single time and in a simple procedure, but TAE performed multiple times is aimed at providing a better effect. Repeated TAE is aimed to achieve a more certain tumor necrotic effect—not just as a single procedure for providing a temporary response but for postoperative long-term outcomes. Recently, the usefulness of drug-eluting beads transarterial chemoembolization has been reported in comparison to conventional TAE, and its preoperative use might be useful [26].

This study had some limitations. First, it was difficult to derive definitive conclusions due to the small number of patients who underwent intended preoperative TAE in this study; further prospective and large-scale trials are necessary to address this. Additionally, there might be some biases in the indication of preoperative supplemental treatment; this depends on the surgeons’ decision and the strategies of TAE followed by the radiologists. Furthermore, advanced HCC has various statuses to be discussed other than tumor size, such as lymph node metastasis [27], and clinical application of biomarker research to clarify the oncological characteristics of HCC is also a future issue [28]. Nevertheless, the results of this study are suggestive of the oncological effectiveness of the intended preoperative TAE for patients with a large HCC.

In conclusion, the large tumor size in HCC is associated with unfavorable outcomes and intended preoperative TAE for large HCC patients (performed multiple times) might improve their prognoses.

## Data Availability

The datasets used and/or analyzed during the current study are available from the corresponding author on reasonable request.

## Abbreviations

HCC: Hepatocellular carcinoma
HBV: Hepatitis B virus
HCV: Hepatitis B virus
NASH: Non-alcoholic steatohepatitis
NBNC: Non-B non-C
TAE: Trans-arterial embolization
AFP: Alpha-fetoprotein
AFP-L3: L3 fraction of AFP
PIVKA-II: Protein induced by vitamin K absence or antagonist II
ICG: Indocyanine green
CT: Computed tomography
IBL: Intraoperative blood loss
RFS: Recurrence-free survival
DSS: Disease-specific survival
RFA: Radiofrequency ablation
